# Thought-Shapers Embedded

**DOI:** 10.3389/fpsyg.2022.918820

**Published:** 2022-07-19

**Authors:** Zsuzsanna Kondor

**Affiliations:** Institute of Philosophy of Research Centre for the Humanities, Budapest, Hungary

**Keywords:** mental representation, public representation, means of expression, cognitive evolution, sensorium, language as thought-shaper, material engagement theory (MET), ocular-centrism

## Abstract

Accepting the idea that the mental representations of concepts, diagrams, relations, plans, etc., are thought-shapers, I suggest going a bit further. Any kind of representation, be it mental or public, i.e., accessible to others, bears thought-shaping potential, albeit not in the same manner. Just as the idea of embodied cognition takes into consideration environmental facilities and obstacles, I suggest investigating thought processes in a broader context, i.e., placing thought-shapers in the context of their formation. I propose that the elements of the above mentioned definition of thought-shapers are built upon a structure that consists of representational skills, means, and institutions. In accordance with the idea of embeddedness and enactment, the need for communication and the given cognitive and physical aptitudes result in different kinds of expression, i.e., different kinds of representations available to others. When an expressional mode solidifies, it opens up new possibilities and limitations. I propose that mundane, almost unnoticeable affordances and their accompanying limits do shape our thoughts thoroughly. In my argument for the thought-shaper potential of the generative technique of public representations, I will delineate a historical overview of representational means in tandem with the main characteristics of different eras’ crucial ideals and patterns of reasoning. I will close the historical overview with a terminological excursion exploring how publicly available representation and mental representation relate to each other and the kinds of ambiguities that accompany the latter term’s use. Accordingly, embedding thought-shapers, I will outline the evolution of different representational techniques and skills. Then, because language is a decisive representational means, I will investigate its orientating and distortive potential. I will rely on some of Bergson’s lesser-known remarks. I will illuminate how ocular-centrism was able to be a decisive metaphor in science and philosophy for long centuries, until recently even. In conclusion, as a case study, I will illuminate how the term “mental representation” as a highly abstract term facilitates and at the same time hinders philosophical and scientific inquiry.

## Introduction

Robert Hanna and Otto Paans define *thought-shapers* as follows: “[b]y ‘thought-shapers,’ we mean mental representations of any or all of the following: allegories, analogies, blueprints, catechisms, diagrams, displays, icons, images, lay-outs, metaphors, mnemonics, models, outlines, parables, pictures, scenarios, schemata, sketches, spreadsheets, stereotypes, symbols, tableaux, and templates” (Hanna and Paans, 2021, p. 1).

Accepting the idea that the listed items’ mental representations are thought-shapers, I suggest going a bit further. Accordingly, any kind of representation, be it mental or public (accessible to others), bears thought-shaping potential, albeit not in the same manner. Just as the idea of *embodied cognition* takes into consideration environmental facilities and obstacles, I suggest investigating thought processes in a wider context, i.e., placing thought-shapers in the context of their formation. I propose that the elements of the above quoted definition of thought-shapers are built upon a structure which consists of representational skills, means, and institutions. In accordance with the idea of *embeddedness* and *enactment*, the need for communication and the given cognitive and physical aptitudes result in different types of expression or representation available to others. When an expressional mode solidifies, it opens up new possibilities and allots limitations.

I am suggesting that not only mental representations have thought-shaper potential, but different techniques for creating public representations also have a considerable effect on the kind of mental representations we can have. Thus I propose that mundane, almost unnoticeable affordances and their accompanying limits do shape our thoughts thoroughly. In my argument for the thought-shaper potential of public representation and its generative technique, I will delineate a historical overview of representational means tandem with the main characteristics of different eras’ crucial ideals and patterns of reasoning.

Because language is a decisive representational means, I will investigate its orientating and distortive potential. I will rely on some of Bergson’s lesser-known remarks. I will illuminate how ocular-centrism was able to be a decisive metaphor in science and philosophy for long centuries, even recently. In conclusion, I will illuminate how highly abstract terms such as *mental representation* can facilitate and hinder philosophical and scientific inquiry. This excursion attempts to demonstrate how representational means can result in abstract terms that are capable of playing a crucial role in different fields of inquiry.

## Representational Means, Chains of Thoughts

In the first part of the article, I will focus on how representational means and institutions affect the intellectual setting (however, I do not endorse the intellectualist approach to cognition). That is, I will investigate how means of expression shape the intellectual focus and interest, and how these means define the accessible and the imaginable. I will not touch upon how moral and political institutions influence our thoughts; the scope of the present paper does not reach beyond the investigation of the interplay between cognitive and bodily skills and the means appropriate to share intentions, thoughts, and ideas. The normative aspect will enter the scene only in the case of objectivity as it emerged in scientific practice to illustrate how technical possibilities shape norms in scientific practice.

Unlike mental representation, public representation is meant to be a representation that is accessible to others. It can take the form of words, signs, gestures, icons, and pictures – just to name a few basic variations. Subsequently, I will not pay much attention to how different institutionalized products of public representation affect the behavior, norms, and goals of the individual, but rather focus on the basic structure’s forming potential of different representational practices changing over time; I will focus on the background facilities which, for example, make an advertisement possible.

The historical overview will show how the patterns of cognition changed in accordance with the emergence of new representational means and methods. I will investigate how different cognitive and bodily skills along the phylogeny paved the way for new representational inventions. Following the line of history, I will first focus on the organizing power of representations as they provide new epistemic access to the environment and cognitive skills, and accordingly, a basis to form community and group-identity. After taking into consideration basic communicational patterns, I will focus on the organization of thoughts in relation to the dominant scholarly representational patterns.

### Representation and Epistemic Access

Within cognitive evolution research, we can find different approaches and strategies (Bender, 2020), and we can differentiate between at least two basically diverse approaches: one that considers cognitive evolution within a representational framework, and another that investigates it in a bodily engagement setting. Here, I will not focus on the question of mental representation, but rather on the effect public representations can have on social organization and cognitive capacity.

Cognitive capacity and development are usually understood in terms of representational skills. Cognitive evolution is an extensively studied field of research within this framework. The questions of what exactly made the evolution of the human mind possible, how language could emerge, and what the pivotal differences between primate and our ancestors’ biological settings were that made the disparate development possible, are hotly debated. Notwithstanding, some pillars of argument are more or less beyond question. The importance of the socially engaged mind of hominids is almost uniformly accepted, and accordingly, the role of communicational needs is considered an important facilitator of cognitive development. Though there are some differences among conceptions of the conditions that were necessary for calling language into being, the importance of language itself is also uncontested.

#### From Brain-Bound Representations to Externalism

The theory of cognitive evolution as Merlin Donald construed it considers cognition in terms of representational skills. Though Donald is committed to representationalism, his theory opened a route toward the idea of the extended and embedded mind. He considers our minds as *hybridizations* where, thanks to the *coevolution* of the brain, culture, and cognition, memory capacity became enhanced by an external symbolic storage system (Donald, 1991, 1993).

In a nutshell, Donald suggests that “the modern human mind evolved from the primate mind through a series of major adaptations, each of which led to the emergence of a new representational system” (Donald, 1991, p. 2). This is an encapsulating process where the cognitive capacities of each stage are incorporated into the next. Accordingly, modern humans possess all earlier representational skills. Donald differentiates three main cultural, and thus cognitive, transitions as each is related to the emergence of a new representational skill. First (i) there was a transition from episodic to mimetic culture when episodic memory was supplemented by mimetic skills. Mimetic skill is a kind of motor skill that allows us to use the whole body as a representational apparatus. Mimesis “was a *multimodal* modeling system” and its output can be voluntarily accessed and retrieved (Donald, 1993, p. 739); it “rests on the ability to produce conscious, self-initiated, representational acts that are intentional but *not linguistic*” (Donald, 1991, p. 168; emphasis added). It is important to stress that unlike the traces in episodic memory, these intentional acts were accessible, visible to others; therefore, they provided the ground for sharing experiences, cooperation, and organization, at least on a basic level. The second transition (ii), from mimetic to mythic culture, is related to the “lexical invention,” i.e., the establishment of language. This required developing the *capacity* for lexical invention, “neural and anatomical modifications for speech” that provide the basis for the further elaboration and extension of lexical and meta-linguistic skills. Interestingly, Donald believes “lexical invention stems from an intellectual need to label whatever thing or relation the mind wishes to ‘capture’ linguistically” (Donald, 1993, p. 780). This lexical invention made further organizational development of groups possible since “[t]he natural product of language is narrative thought … a method of modeling reality” (Donald, 1993, p. 745). Accordingly, increasingly refined social customs and institutions could be built, allowing for an increasingly well-defined identity.

Donald suggested “blending language onto a larger mimetic behavioral framework” (Donald, 1993, p. 781). This idea has been refined in many ways by now, but let me refer to a suggestion that illuminates an important element which is unavoidable for linguistic representation. If we accept that gesture came first and hominid ancestors were social-minded as many suggest (Donald, 1991; Dunbar, 1998), there is a need for a bird’s eye perspective. If “there is collective action with role specialization, and if agents do not always act in the same role (if sometimes I act to drive the game; if sometimes I am the lookout; if sometimes I wait in ambush), then each team member does need a bird’s eye representation of the collective activity. But bird’s eye representation is a crucial representational capacity needed for syntax. It is the distinction between a role and the occupant of that role” (Sterelny, 2012, 2147). That is, at a higher level of organization there is a need for meta-level distinctions between the role and the player of the given role, and the ability of cooperating in a rather strictly orchestrated manner keeping in mind the end-goal and the necessary steps to reach it. Hence, collective activity levels up to a planned collectively, and at the same time, individually, controlled process. The mates’ behavior now operates under a new light: they became part of an orchestrated play.

The third transition (iii), from mythic to theoretical culture, came about when the increasing memory-load could be alleviated by the emergence of an external symbolic storage system. “At this point, since the Upper Paleolithic, there emerged the *visuosymbolic invention*, then entered the scene the use of *external memory* (in this process the so-called literate class played an important role, and started to take the governance), and finally arose “externally nested cultural products,” viz., *theory* (Donald, 1993, p. 745). In this paper I do not intend to go into detail regarding what ambiguities can be found in Donald’s theory as regards to the relation between visuosymbolic and lexical inventions, rather I will emphasize that in Donald’s conception, cave paintings are considered as the first steps toward an external symbolic storage system. As he wrote “the first pictorial images themselves were also external representations. They existed outside of the individual, rather than in visual memory. Therefore a technological bridge was under construction that would eventually connect the biological individual with an external memory” (Donald, 1991, p. 284).

#### Material Engagement and Epistemic Access

Donald’s reliance on the social brain hypothesis (Dunbar, 1998) reconstructed human cognitive evolution in terms of representational skills. Representational skills play a decisive role in social organization. In his conception, mimesis provides “the missing link, a necessary preadaptation for language” (Donald, 1993, p. 778) and the traces of first visual artifacts are considered the first elements of a technological bridge between biological and external memory, paving the way for new representational and organizational inventions. Subsequently, I will briefly review *material engagement theory* (MET), which illuminates how archeological findings can help reconstruct cognitive turning-points, and significantly, how material artifacts modify epistemic access to the environment.

Like Donald’s theory, *neuroarchaeolog*y operates in terms of a close relation between culture and mind, but in a rather active manner. It is “strictly an interactionist approach, aiming primarily to understand the bidirectional links between brains, minds and culture” (Malafouris, 2010, p. 64). That is, cognitive activity is not dedicated to modeling the world out there, but rather it manifests itself in different kinds of bodily activities. Consequently, these engagements form not only the environment but at the same time reveal different aspects and possibilities for further interactions.

The criticism of building upon mental representational skills in cognitive evolution is quite obvious if we think of the accessible evidence for archeology. That is, against the background of material artifacts and fossils, “mentality defined on the basis of brain-bound cognitive processes and genuine ‘non-derived’ representations” (Malafouris, 2013, p. 37) is hardly achievable. It seems more promising to start with material traces in relation to bodily skills, i.e., on the basis of material findings and analogies of craftsman-like activities following the route of “think[ing] through, with, and about the material world” (Malafouris, 2013, p. 34). Accordingly, from an archeological point of view, enactivism seems to be a fruitful starting point.

Though Donald thinks bodily skills are important even in the evolution of vocalization, he does not take into consideration the consequences of interaction with the environment, i.e., the dynamically altering experiences along the process. “The mind works through the body. To localize it exclusively within the brain is not strictly correct. Moreover, we often think not only through the body, but beyond it” (Malafouris, 2013, p. 33). That is, the primordial interaction with the world happens through the body, and via different bodily skills new behavioral patterns can emerge out of interaction. The term *creative thinging* highlights the capability of “inventiveness that is inseparable from the capacity to affect and be affected through movement and sensation from the phenomenal qualities of the materials that surrounds us” (Malafouris, 2014, p. 144). Accordingly, inventions entail new experiences and hence, affordances^1^ for further inventions.

Each invention provides a new perspective toward a certain phenomenon and even toward our own capabilities. Early cave paintings, for example, reveal special characteristics of the depicted scenario. Beyond the invention of different techniques of depicting visual scenery, such as mapping three dimensions onto two, highlighting certain aspects in accordance with the intended emphasis, or making a dynamically altering scene static, there are important changes as regards the perception of the capability of creating pictures. “Through the process of ‘imaging’ the underlying mechanisms of human perception are being transformed to an object *for* perception and contemplation. … The Paleolithic image-maker constructs an external scaffold that affords the world to be seen and experienced in ways that the physiology of the naked eye by itself does not allow. This scaffolding also enables a new direct understanding of the human perceptual system and thus offers to the Paleolithic individual the opportunity to become in some sense, maybe for the first time, the engineer of his or her own perception. The image, as it is also the case with language, enabled humans *to think about thinking”* (Malafouris, 2007, p. 299f).

That is, in terms of bodily activity and affordances the things of the environment enter a new light and previously unnoticed aspects become visible thanks to active engagements. These changes and discovered affordances induce new kinds of engagements. In this sense, public representations are important facilitators of cognitive development not exclusively in terms of representational skills, but also in terms of epistemic access and affordances. However, these inventions allot hindering effects as well: consider problem-solving when the accustomed routes hamper new approaches, or how paradigm-shifts resolving an anomaly are only possible under a radically different set up.

### Representation and the Organization of Thoughts

Whether from a representational or an enactive^2^ point of view, cultural artifacts have a considerable effect on a social-organizational and cognitive-epistemic level; they are capable of inducing certain changes in “the use patterns of the brain” (Donald, 1991, p. 14) and creating new affordances. First, I will recapitulate the thought-organizational patterns of so-called *oral cultures* where written records were unknown; then I will trace the changes after the emergence of alphabetical writing and other inventions in the era of so-called *literacy*.

#### The Noetic World of Orality

It is really hard to imagine not being able to make notes or write messages, having access only to our own biological memory and that of community members as well as some external scaffolds in terms of mnemonics (Yates, 1984). Cultures without script entail organizational, behavioral and communicational patterns fundamentally different from ours. Hence the relation to the past, to time, to the community and its members, as well as the importance of experience both on a communal and individual level are radically divergent as compared with the era of the literate mind.

“The word is something that happens, an event in the world of sound through which the mind is enabled to relate actually to itself” (Ong, 1981, p. 22). That is, words provide a certain epistemic access to the subject matter that the talk is about, and as they are audible “[a]s events, words are more celebrations and less tools than in literate culture” (Ong, 1981, p. 31). We can have in mind the Heideggerian criticism of Western culture and philosophy because it depleted and deteriorated words, therefore they lost their power to call things into being; they became mere instruments and signifiers (Kondor, 2008, p. 106–108; Kondor, 2016, p. 136f).

Because of the load on memory there were various techniques to help recollection. Thoughts were “mnemonically structured” (Ong, 1981, p. 30), i.e., memory was helped by formulaic expression, rhythm, and even kinesthetic scaffolding^3^. To use Havelock’s term, we can consider a *storage language* (Havelock, 1986, p. 59) that is not subordinative, but rather additive. It is redundant, helping to follow the story line; aggregative but not analytic; and conservative as new elements of a story can never entirely displace the old ones. The topics at issue and the concepts are in very close relation with everyday life, i.e., abstract and neutral concepts are unaccounted for. The means of verbalization is agnostic, constructing a narrative which will aid the speaker, more precisely, the *rhapsodos*, and its audience in preserving knowledge. This way of expression is empathetic and participatory, and thus entails a communal commitment to the known. Storage language had to go through a thorough process of alteration before it could fulfill the requirements of written language.

#### Mindset in the Age of Literacy

According to Ong’s terminology, after the invention of written records, especially alphabetical writing, the organization of thoughts and the social institutional setting gradually transformed. From there on, we can talk about literacy (Ong, 1983). As alphabetical writing spread and gradually became customized, the abovementioned technical limits faded away, i.e., since the memory-load decreased, formulaic composition vanished; neutral and abstract concepts gradually proliferated. Though writing appears to be a mere technique, and as Ong many times emphasized, it “represents sound,” i.e., the acoustic phenomenon of the word, it paved the way toward new needs. The orienting situation, gestures, and facial expressions had to be expressed by words: a need emerged for situation-independent terms for describing a situation understandable in the long-run for the people who are not involved in it. If we think in long-term scenarios, we need general subjects instead of the name of famous heroes or well-known personalities. As the coercive power of the here and now dissolved, more and more abstract concepts entered the scene. The floor was open to create a universe of concepts not directly related to the here and now and actual practical needs. The past entered as a bygone fact recorded in texts and its vivid relation with the present moment vanished; therefore, its regulative force gradually altered: regulation and organization became anchored in legislative texts.

“Writing heightens consciousness. Alienation from a natural milieu can be good for us and indeed is in many ways essential for full human life. To live and to understand fully, we need not proximity but also distance. This writing provides for consciousness as nothing else does” (Ong, 1983, p. 82). And this process gained reinforcement by additional technical invention, viz., the printing press.

#### The Printing Press

The emergence of the printing press also induced important changes in scholarly and everyday practice. At the very beginning, the applied patterns were very similar to those of written script. “In fact printed books were first hardly distinguishable from manuscripts” (Eisenstein, 1979, p. 32, 51). However, differences in appearance emerged thanks to the effectiveness of this new technology of text-production, with a long-lasting effect on intellectual life. Dialogs had been transformed into linearly constructed argumentations even more detached from the world of life; printed texts were easier to read because complicated abbreviations ceased to be used, and words were clearly recognizable. Reading became quicker; and the possibility of duplicating images made it possible to use pictures as well as words or concepts.

All these modifications called new habits in intellectual life into being. After the spread of printed texts, the practice of loud reading dropped slowly into oblivion and abdicated its place to silent reading. Earlier, writing was a “solipsistic operation” (Ong, 1983, p. 101) and now reading had become solipsistic too. As printed books and libraries of these printed texts can be considered as “invisible cathedrals of memory of the past” (Eisenstein, 1979, p. 66), and “[p]rint encloses thought in thousands of copies of a work of exactly the same visual and physical consistency” (Ong, 1983, p. 132), printed texts suggest a kind of “self-containment”. While individual engagement gained more and more weight in scholarly practice, with the heritage of the past becoming accessible to the individual, reasoning became more and more solipsistic; accumulating knowledge seemed to be the result of individual effort.

The possibility of duplicating images, i.e., to use pictures as definitions, leads to a blossoming of science, especially those sciences which earlier were not able to accumulate knowledge because of the necessity of particulars, as in the case of botany (Ivins, 1953). Importantly, the apparently merely technical invention of the printing press entailed a key shift in focus from generalities toward particulars^4^.

Along with the individualization of scholarly work, collaboration between different experts emerged: “fruitful forms of collaboration brought astronomers and engravers, physicians and painters together, dissolving older division of intellectual labor and encouraging new ways of coordinating the work of brains, eyes and hands” (Eisenstein, 1979, p. 56). Additionally, scholars previously “had to engage in ‘slavish copying’ of tables, diagrams and unfamiliar terms” but print technology “produc[ed] a new situation which released time for observation and research” (Eisenstein, 1979, p. 47). That is, print technology with seemingly slight technical modifications, transformed scholarly activities and facilitated the shift of interest: particulars gained importance. This process is nicely traceable in the modification of scientific ideals.

#### The Epistemic Value of Objectivity

Because words were easily duplicable and separation from the actual situation required general terms, ancient Greeks, and even “reasonable” people of the 18th and 19th century, lacked the proper technology with which they could handle and take into account particulars; so they thought in “generalities” (Ivins, 1953, p. 91)^5^. The ease of “the exactly repeatable and therefore seemingly permanent verbal formula” was for a long time a decisive aspect in scholarly activities. With the invention of the printing press, images as definition-like repeatable items gave an impulse of particulars in scholarly practice, but other difficulties soon emerged: how can the reliability of scientific works be satisfied? When both general concepts and particular cases are accessible, how can we guarantee the authority of science? Daston and Galison’s (2007) careful investigation of the modification of objectivity from the 18th to the early 21st centuries nicely shows the relation between technical facilities and the ethos of scientific inquiry. The ‘‘nebulous notion of objectivity’’ took shape as a result of investigative practices and their results as embodied in scientific atlases^6^. The authors consider the scientific atlas “as a touchstone to reveal the changing norms that govern the right way to see and depict the working objects of science” (Daston and Galison, 2007, p. 49).

They differentiate three ideals as regards objectivity. *Truth-to-nature*, or the “viewpoint of angels” (Daston and Galison, 1992, p. 82) suggested depicting its object as “an idealized, perfected, or at least characteristic exemplar of a species or other natural kind”. To reach this generalized image atlas-makers “carefully selected their models, … smoothed out anomalies and variations in order to produce what we shall call ‘reasoned images.’ They defended the realism … of underlying types and regularities against the naturalism of the individual object, with all its misleading idiosyncrasies” (Daston and Galison, 2007, p. 42).

The mid-nineteenth century saw the emergence of *mechanical objectivity*. This non-interventionist approach suggested the “self-discipline of saints” (Daston and Galison, 1992, p. 82), i.e., as little modification in depiction as possible, and accurate reproduction of the given exemplar of a specimen. This ideal was in tandem with the conviction that “machines were paragons of certain human virtues” such as patient, strenuous, and ceaselessly alertness and accuracy (Daston and Galison, 2007, p. 123).

Mechanistic objectivity was reinforced by the invention of photography and the X-ray. However, the X-ray provided grounds for dubiety quite early in medical practice since certain distortions and/or spurious juxtapositions could be intentionally produced. But there was an even more significant difficulty: the amount of accessible and recorded individual cases could not answer the question of “how can one distinguish between variations within the bounds of the ‘normal’ and variations that transgress normalcy and enter the territory of the pathological?” The solution was upgrading the rare striking deviations. These deviations were considered as “boundary posts of the normal” (Daston and Galison, 2007, p. 309). *Trained judgment* became an important part of scientific practice: beside mechanically objective images the need for an “interpretative eye” emerged which, thanks to its expertise, could distinguish between normal and pathological cases. Therefore, in the first half of the twentieth century trained judgment gradually “became a new kind of regulative ideal” capable of reshaping expectations as regards scientific performance (Daston and Galison, 2007, p. 321).

We should note here that *structural objectivity* from the late nineteenth and early twentieth centuries “waged war on images in science” (Daston and Galison, 2007, p. 45). Its proponents were mainly mathematicians, physicists, and logicians who aimed at general structures as opposed to the particulars of nature.

Objectivity is understood as a “method of understanding” by which distortive elements can be reduced or eliminated (Daston and Galison, 2007, p. 51). However, “how things seem to us depends both on the world and on our constitution”; hence, we need to know our relation to the world (including the formation of our past and present experiences and conceptions). That is, “objectivity allows us to transcend our particular viewpoint and develop an expanded consciousness that takes in the world more fully” (Nagel, 1986, p. 5). The idealized depiction of specimens in accordance with the *true-to-nature*, the accurate depiction of a specimen as *mechanic objectivity* suggests, and the pure structure of nature without its particular details all embodied an attitude which relied on an accessible technique of depiction, and an ideal of how reality can be reconstructed most accurately. We can recognize the traces of convictions entailed by philosophical considerations: “whereas the self-restrained by mechanical objectivity was largely the creation of will-centered post-Kantian philosophy, that renounced by structural objectivity was in part the discovery of science itself, particularly the then young sciences of sensory physiology and experimental psychology” (Daston and Galison, 2007, p. 258). As we can see, the acceptance and application of each ideal entail anomalies. Both the distortive potential of the individual intervention and the vast amount of slightly different individually depicted cases raise unsolvable questions within the accepted paradigm. Also, structural objectivity’s alternative scarified perceptual experiences, gave up “one’s own sensations and ideas in favor of formal structures accessible to all thinking beings” (Daston and Galison, 2007, p. 257), thus entailing the challenge of solipsism.

### Criticism and Virtues

From the time of the emergence of alphabetical writing we can find traces of nostalgia and anxiety as regards the imminent changes implicated by the new technology of public representation. Consider Plato, regarded as a philosopher of the transition from orality to literacy. He is “aware that he is engaged in a process of ‘naming names,’ fixing them, we might say, as new names, new insofar as they are to become symbols of conceptual identities” (Havelock, 1978, p. 327). But at the same time he was dubious as well: he thought that writing would destroy memory and weaken the mind (Plato, 1997, *VII, Letter* 341d); that true knowledge could be preserved and conveyed exclusively by the soul (Plato, 1997, *Phaedrus* 278a); and that because written words bear a thing-like character, they suggest exactly the opposite. Lycurgus, who was afraid of writing, made legislation externalized (Pattison, 1982, p. 56). Later, in the 13th century, Thomas Aquinas wrote that “[o]nly hearing can fully be believed” as opposed to texts (Pattison, 1982, p. 78).

As print technology became an obvious part of scholarly work stripped from the remedies of manuscripts and silent reading vanished, hindering effects and ambiguities of linguistic expression emerged. Consider Bacon’s idols of the Marketplace, i.e., as he formulated “Men associate through talk; and words are chosen to suit the understanding of the common people. … The definitions and explanations with which learned men have been accustomed to protect and in some way liberate themselves, do not restore the situation at all. Plainly words do violence to the understanding, and confuse everything; and betray men into countless empty disputes and fictions” (Bacon, 2000, p. 42). In the second half of the 17th century, Leibniz explicitly expressed his demand for a universal, less ambiguous language and thought Chinese characters (as opposed to alphabetical writing) would be the prototype for an ideal philosophical system of notation, free of the ambiguities of our spoken language and directly correlated with the objects of thought without the distorting intermediacy of spoken language (Pattison, 1982, p. 34). Both Bacon and Leibniz talk about the harm caused by spoken language which is supposed to be less accurate, neat, and eximious than required for science and philosophy. We can see that, as opposed to the mind of late orality and the early times of literacy, scholarly work was thought to be a sterile and accurate system where the inappropriateness of words and concepts are painfully hindering.

In the 19th century, a criticism of scientific elitism and the one-sided intellectualist approach to the human mind emerged. Nietzsche contrasted the magnificence of ancient poetry with the pale, abstract and inanimate writings of his time; Bergson (as we will see) highlights the importance of intuition in relation to the intellect as regards the possibilities provided by language; and a bit later, Heidegger thoroughly criticized Western metaphysics as it distorted the original meaning of important concepts such as *logos* and *aletheia*, to name those which are in direct relation to language and literacy. In the age of *secondary orality* the emergence of different technological inventions (such as photography, film, the gramophone, radio – to name a few), create situations which perform similarly to some institutions of primary orality such as “participatory mystique, its fostering of communal sense, its concentration on the present moment, and even in its formulas” (Ong, 1983, p. 136). This direct relation to everyday life and the quest for the primordial immediacy of experiences took form in Heidegger’s criticism of culture and metaphysics, and even quite explicitly new technology. Heidegger believed there was a direct immediate relation to *Being*, and this immediacy could be expressed by spoken language and by the hands. That is, “[t]he word indicated by the hand and appearing in such marking is writing… Being, word, gathering, writing denote an original essential nexus, to which the indicating-writing hand belongs. In handwriting the relation of Being to man, namely the word, is inscribed in beings themselves” (Heidegger, 1998, p. 84f). Setting, pressing, and printing are all the pre-form of the typewriter as these activities distort and mechanize this relation. Heidegger was radically critical as regards the radio, e.g., it provides information but because it makes things seemingly accessible from afar it makes perception and thinking shallow.

In *Being and Time* he proposed that although language can be investigated as a means of expression, primordially it has a revealing potential, it can call things into being and accurately indicate how we relate to our world. “Nevertheless, the ultimate business of philosophy is to preserve the *force of the most elemental words* in which Dasein expresses itself, and to keep the common understanding from leveling them off to that unintelligibility which functions in turn as a source of pseudo-problems” (Heidegger, 1962, p. 262). Heidegger’s criticism of Western metaphysics is partially based on the restoration of some elementary words as he illuminated the gradual distortion of *logos* becoming logic^7^, and *aletheia* becoming *veritas.*

But, as Heidegger believed, thinkers and poets can preserve the force of language because they relate to it in a particular, though slightly different way. Philosophers “show the work of *aletheia* in its entirety, i.e., as concealment and un-concealment,” while the poets’ task is to highlight unconcealment in a peculiar way. Poets use words as signs as opposed to considering them as a means of designation, mere signifiers. Hence, the words of a poem can call things that they name to presence. Furthermore, poems speak in images. “This is why poetic images are imaginings … that are visible inclusions of the alien in the sight of familiar” (Heidegger, 2001, p. 223). Accordingly, poems can illuminate some aspects of the world unseen by ordinary sight with the help of and through the accustomed scenery. That is, there is a need for a renewal in language and the relation to the world, and this can be reached rather with the help of art.

## The Relevance of the Sensorium

We can think about cultures in terms of *sensorium*. “By the sensorium we mean here the entire sensory apparatus as an operational complex” (Ong, 1981, p. 6). The importance and the dominance of visual experiences were recognized quite early: the pre-Socratic Simonides of Ceos (Approx. 556-468 B.C.) was an admired poet of ancient Greece who considered painting as silent poetry and poetry as painting that speaks (Yates, 1984, p. 28). That is, both the painter and the poet rely on visual imagery, and mnemonics are also related to visualization^8^. He championed the supremacy of sight over other modalities.

Ancient cultures varied greatly as regards senses – they relate different modalities to conceptual processing. “The Hebrews tended to think of understanding as a kind of hearing, whereas the Greeks thought of it more as a kind of seeing, although far less exclusively as seeing than post-Cartesian Western man generally has tended to do” (Ong, 1981, p. 3). Similarly, ancient Greeks relate geometry to tactile experiences, unlike modern Western scholars. The Greeks “thought more about the way various shapes felt (they tended to imagine themselves fingering their way around the geometrical figure),” their geometry was that of the *participator*, while ours is that of the *spectator* (Ong, 1981, p. 4). Similarly, in the so-called *verbomotor* cultures where rituals involve the whole body, recitation is kinesthetically supported.

Even in ancient oral cultures where live intercourse was the main source of knowledge and any kind of exchange, mnemonics and the formulaic-rhythmic-artistic way of expressing deeply embedded current and past worldly experiences encourage thinkers recognizing the importance of sight. After the invention of the alphabet, “[t]hough words are grounded in oral speech, writing tyrannically locks them into a visual field forever” (Ong, 1983, p. 2). Although alphabetical writing probably has its roots in pictograms it lost its connection with things, and it rather represents acoustic sound (Ong, 1983, p. 91). The visualization of the acoustic phenomena could facilitate the importance of sight as compared with other modalities and even those philosophers who were against writing, like Plato^9^, expressed their thought with the help of visual metaphors as in the case of the allegory of the cave. Later on, as well, either through the eye of the body – as empiricism suggested – or by the mind’s eye – as rationalism suggested – vision was considered as helping us in mirroring reality (Engström and Selinger, 2009, p. 29).

In a nutshell, the printing press solidified the spatialized character of conceptual work and later, telecommunicational means first mediated sound, and soon afterward the visual scenario as well. In the age of digital technology, first the technically easy production of texts was followed by acoustic and visual simulations. The immersiveness of virtual reality heavily relies on spatial experience provided by sight. That is, while science is becoming an increasingly important thought-shaper of everyday life, despite new technologies of communication and dissemination, its results emerge spatially bounded. Even new imaging techniques of neurology result in printed or displayed images and/or diagrams. That is, the importance of sight, and ocular metaphors have been dominant from very early times on, already evidenced in pre-Socratic times.

Subsequently, I will attempt to explicate how the dominance of space in intellectual exchange resulted in a peculiarly one-sided way of thinking. We can call this kind of thinking *intellectualism* (a recently hotly debated and criticized approach in the literature of embodied cognition), or use the Heideggerian term *calculative thinking*, which became dominant and overcame meditative thinking^10^.

## Language as a Thought-Shaper

As we can see, ocular-centrism together with alphabetical writing, then printing, and finally digital technology bound the word greatly to vision and spatialized it^11^. That is, both concrete and highly abstract concepts entered into the space first set in stone, later written, and then printed on paper, and more recently on the screen. This spatially bound character of the terrain of mental/intellectual engagement has far-reaching effects on our way of thinking.

### Why Thoughts Spatialized?

As Henri Bergson^12^ at the end of the 19th century concluded: “The mistake of ordinary dualism is that it starts from the spatial point of view: it puts on the one hand matter with its modifications in space, on the other unextended sensations in consciousness. Hence the impossibility of understanding how the spirit acts upon the body or the body upon spirit” (Bergson, 1911, p. 294f)^13^. He suggested starting with perception. Perception in its pure form belongs to the material world, but as conscious beings we have access not only to spatially determined states, but to processes stretched in time. That is, in the world of matter, in space, there is “mutual externality without succession,” whilst “within our ego there is succession without mutual externality” (Bergson, 2001, p. 108). Accordingly, only conscious beings can perceive succession in time; mere material things are only in a single state at each moment with no regard either to the earlier or the later state. In contrast, for humans a given state of affairs is always linked to past experiences and reactions in the future because in accordance with our past memories, our (re)action is to be executed in the (imminent) future.

Bergson suggests that a spatially bound way of thinking is not by chance. Because humans are both spiritual and physical beings and their access to the physical world is provided by different sense organs (that are actually also physical), the conceptual apparatus evolved in accordance with the physical. We need to live and survive in a physical world; therefore, we need means that are in accordance with this world. “*Primum vivere*. Memory, imagination, conception and perception, generalization in short, are not there ‘for nothing, for pleasure.’ … it is because they are useful, because they are necessary to life” (Bergson, 1946, p. 60). Humans have the privilege of free will, and free will necessitates the possibility of choice. Choice is possible because consciousness and memory create a framework within which we can reach beyond the spatially determined matter of here and now and stretch the moment in time^14^.

Language additionally provides us a conceptual apparatus that can facilitate organizational work within the material world and make use of material goods. But although humans are capable of connecting different phenomena in time, spatial arrangements of things are decisive in thinking and conceptual manipulation. As Bergson put it: “[t]here is a real space, without duration, in which phenomena appear and disappear simultaneously with our states of consciousness. There is a real duration, the heterogeneous moments of which permeate one another; each moment, however, can be brought into relation with a state of the external world which is contemporaneous with it, and can be separated from the other moments in consequence of this very process. The comparison of these two realities gives rise to a symbolical representation of duration, derived from space” (Bergson, 2001, p. 110).

### Language Spatialized

Bergson believes that “intelligence is the prolongation of our senses. … If the intellect has been made in order to utilize matter, its structure has no doubt been modeled upon that of matter” (Bergson, 1946, p. 42). That is, though humans are spiritual, they are at the same time material beings in close relation with the material world. To be able to manage this world, we need intellectual skills which are supported by language. Language is efficient because it is in accordance with matter, it is tailored in spatially determined relations.

Things in space are clearly separated from one another – they have shape, color, etc., and are enclosed by surfaces; their relations are grasped in terms of physics. Seemingly, our language quite aptly captures these spatially determined features of our environment. But our thinking has become assimilated to space. This was clearly demonstrated quite early by the so-called Zeno paradox. Zeno of Elea “by drawing attention to the absurdity of what he called movement and change, led the philosophers – Plato first and foremost – to seek the true and coherent reality in what does not change” (Bergson, 1946, p. 165); “metaphysics was led to seek the reality of things above time, beyond what moves and what changes, and consequently outside what our senses and consciousness perceive. As a result it could be nothing but a more or less artificial arrangement of concepts, a hypothetical construction” (Bergson, 1946, p. 16). That is, the case of Achilles and the tortoise nicely exemplifies how distortive it is when we try to grasp motion in terms of space. Spatial arrangement is in accordance with separateness and differentiation, but these characteristics are stable; movement is considered as infinitely dividable units of stable states placing them beside each other. “A movement could not alight on an immobility for it would then coincide with it, which would be contradictory. The points are not *in* the movement as parts, nor even *under* the movement as places of the mobile. They are simply projected by us beneath the movement like so many places where, if it should stop, would be a mobile which by hypothesis does not stop. They are not, therefore, properly speaking, positions, but suppositions, views or mental viewpoints” (Bergson, 1946, p. 212f).

Beyond the inappropriate framework of apprehending motion and change, there are some additional mechanisms in concept-formation: generalization and abstraction. These tendencies are characteristic even at a basic level of living organisms. Because every tissue behaves in accordance with its needs, “it isolates the characteristic which interests it, …; it classifies, and consequently abstracts and generalizes. Doubtless, in almost all cases and probably in all other animals except man, abstraction and generalization are actually experienced and not thought” (Bergson, 1946, p. 61).

Because abstraction and generalization are actually *thought* by humans, the distortive potential of concepts are strengthened. When objects are examined we notice features common in some of the items – we compare the objects having common features. “But as the comparison has brought out a resemblance, and as the resemblance is a property of the object, and as a property seems very much as though it were a *part* of the object possessing it [but it is not!], we are easily persuaded that by juxtaposing concepts to concepts we shall recompose the whole of the object with its parts and obtain from it, so to speak, an intellectual equivalent” (Bergson, 1946, p. 195). This procedure suggests that if we line up common features of a phenomenon, we gain its representation. This happened in the case of duration: by aligning the concepts of unity, multiplicity, continuity, infinite divisibility, etc., we expect to get the representation of duration. But it is an illusion and a danger at the same time (Bergson, 1946, p. 195). Additionally, if a concept symbolizes a particular property because this property is considered being common to an infinity of things, “it always more or less distorts this property by the extension it gives to it” (Bergson, 1946, p. 195f). Therefore, when we attempt to reconstruct a phenomenon with concepts, we use these concepts as if they would be clear and definite, but as we extend their scope they definitely loose clarity. Because concepts are based on abstraction and extension they considerably decrease their accuracy.

Similarly, when we describe a simple sensation, the description suggests that the quality of the sensation, e.g., the taste of a flavor, is something stable, a solid quality. “But in reality there are neither identical sensations nor multiple tastes: for sensations and tastes seem to me *objects* as soon as I isolate and name them, and in the human soul there are only *processes*” (Bergson, 2001, p. 131). Bergson calls attention to the fact that words are capable of modifying our sensory experiences as a means of social intercourse: some common convictions may “[cover] over the delicate and fugitive impressions of our individual consciousness” (Bergson, 2001, p. 133), i.e., by words it is possible to overwrite certain sensations, impressions.

In sum, words and concepts solidify qualities, thus suggesting that perception or any conscious state can be reconstructed by aligning well-defined concepts; but a concept cannot be well-defined because of the process of generalization and abstraction. The recognition of these ambiguities obviously leads philosophical inquiry to demand a new perspective and terminology describing mental phenomena. Nevertheless, loose concepts and ‘‘loose grammar’’ can easily result in pseudo-problems or dead-ends^15^.

### Intuition and Metaphoric Language

Bergson illuminated how spatially organized language leads us astray. He demonstrated that an ambiguous conceptual apparatus tailored in accordance with spatial relations raised the illusion of psychic life as something which can be reconstructed with the help of deliberately constructed concepts. “The state, taken in itself, is a perpetual becoming. I have extracted from this becoming a certain mean of quality which I have supposed invariable: I have thus constituted a state which is stable, and by that very fact, schematic.” Accordingly, scientific analysis will operate on immobility. But “the real, the actual, the concrete” can be recognized “by the fact that it is variability itself” (Bergson, 1946, p. 211f).

In order to have a grip on the real, we need to rely on intuition as well as on intellect. Bergson was aware of the limits of expressive power. That is, he clearly knew that the language we use is deeply integrated, and beside its disadvantages it is only possible to overcome its limits with some restrictions. “We necessarily express ourselves by means of words and we usually think in terms of space. That is to say, language requires us to establish between our ideas the same sharp and precise distinctions, the same discontinuity, as between material objects” (Bergson, 2001, p. XIX).

Bergson believes that although science and metaphysics differ in their methods and subject, “they can both touch the bottom of reality” (Bergson, 1946, p. 41), and there is no difference between their values and source of experience. When he suggests that metaphysical investigation should rely on intuition he is aware of the fact that “[i]ntuition will be communicated only by the intelligence” (Bergson, 1946, p. 47), but because “to think intuitively is to think in duration” (Bergson, 1946, p. 37), then intuition “signifies first of all consciousness, but immediate consciousness, a vision which is scarcely distinguishable from the object seen, a knowledge which is contact and even coincidence” (Bergson, 1946, p. 35). With intuition, we can recognize the straitjacket of intellect as it starts with the immobile and attempts to reconstruct movement and “sees in immobility only an abstract moment, a snapshot taken by our mind, of a mobility” (Bergson, 1946, p. 38).

Notwithstanding, creating a new conceptual apparatus based on intuition is not an obvious enterprise. We are addressed to imagery: “there are cases in which it is imagery in language which knowingly expresses the literal meaning, and abstract language which unconsciously expresses itself figuratively. The moment we reach the spiritual world, the image, if it merely seeks to suggest, may give us the direct vision, while the abstract term, which is spatial in origin and which claims to express, most frequently leaves us in metaphor” (Bergson, 1946, p. 48).

If we take into consideration the basic criticism of a spatially determined conceptual framework as Bergson construed it, as well as the Ongian considerations about spatially locked words brought about by literacy, and the Heideggerian caveat as regards the elementary force of words, i.e., having the power of calling things into presence, we can see how radical the change that spatialization of the word induces is. Concepts are tailored in accordance with space and additionally are recorded by writing, covering the primordial relation to the world suggesting fixed and separated qualities of experiences, and an external relation to the phenomena they refer to. In Ongian terms, with the transition from literacy to the age of secondary orality we can see efforts to overcome the traditional conceptual framework (like at the beginning of literacy). Creating a new vocabulary as Bergson suggested, establishing a fundamental ontology restoring the power of elementary words, or providing access to the primordial structure of consciousness and cognition as phenomenology suggested, are important steps toward setting the stage for the different approaches of so-called embodied cognition and preparing a paradigm-shift in cognitive psychology as well.

## A Thought-Shaper Concept: Mental Representation

In the last section of the present paper, I will delineate how the term “mental representation” was formed, and then, underlining its polymorphic character, I will illuminate the diverse usage of it in relation to the peculiar character of concepts as Bergson and later Wittgenstein called our attention to it. With this short overview of the terminological peculiarity of the state of affairs as regards the usage of the term, I hope to cast light upon how the concept of mental representation shapes inquiry, and therefore our thoughts; and to illuminate that spatially based conceptual framework and analytic thinking can result in ambiguous, sometimes misleading concepts.

### The Idea of Representation

Representation and isomorphism belong together, though this relation was not uniformly accepted. Isomorphism can be “any degree or kind of resemblance, likeness, or similarity of pattern, structure, or relational organization between entities or events as defined in the broadest sense” (Watson, 1995, p. XI). Though Descartes himself denied the necessity of resemblance between the represented entity and its idea, he stays on the route of isomorphism because in the description of vision he maintained a point-by-point isomorphism between the seen object and the pineal glad’s vibration, thus the idea of this object. Nevertheless, even with Descartes we can notice some ambiguities in the usage of the term *idea* as highlighted by Descartes himself.

In his *Meditations* he referred to a kind of ambiguity as regards the term *idea*. “Idea can be taken materially, as an operation of the intellect … [and] can be taken objectively, as the thing is represented by that operation” (Descartes, 1995, p. 7). In another piece he calls attention to the difference “between the (i) sensation we have of light (i.e., the idea of light which is formed in our imagination by the mediation of our eyes) and (ii) what it is that produces this sensation within us (i.e., what it is in a flame or the sun that we call by the name ‘light’)” (Descartes, 1985, p. 81). That is, Descartes distinguishes between (i) the sensation of light which is mediated by our eyes and processed further in a way and (ii) what is the cause of our sensation. This distinction brings to mind William James’ caveat according to which we often unnoticingly make double the same phenomenon: once we count it “[a]s ‘subjective’ we say that the experience represents; as ‘objective’ it is represented. What represents and what is represented is here numerically the same; but we must remember that no dualism of being represented and representing resides in the experience *per se*,” or “being Gedanke and Gedachtes, the thought-of-an-object and the object-thought-of” (James, 1987, p. 1151).

The special character of language as Bergson suggested makes all phenomena we are referring to object-like, and hence the same phenomenon easily oscillate between different senses: the idea of light can be considered as a result of a given *mental act*; can be a *representation of something* that makes things visible, i.e., we call light a certain luminosity; and at the same time it can be the *cause of the mental act* itself: we can see things in a room because of adding some light. This ambiguous situation is still characteristic if mental representation is in question.

### Science and Representation

According to psychology and cognitive science, mental representation is indispensable when mental phenomena are to be explicated; it plays a role in the reconstruction of perception, remembering, reasoning, and dreaming – to mention a few mental activities. Cognitive science maintains that representational states are content-bearing and carry information in a certain way. We perform operations over them and these operations result in problem-solving and planning. Representations presuppose certain relations, such as mapping, (i.e., environmental structures are coded onto internal structures in accordance with certain rules); intentionality, since representations are about something; asymmetry between the representation and the represented; and standing-in relation, i.e., that which a representation stands for is the representation’s content.

In scientific literature, representation is understood as based on isomorphism between the representation and the thing that is represented. It can be considered as “a theoretical object that bears an abstract resemblance to something outside itself. …We can think of knowledge, percepts, images, plans, intentions, and memories as representations” (Baars, 2011, p. 41). And it is quite explicitly expressed that there is no clear empirical evidence that mental representation does exist. Rather, in “psychology we often infer that human beings have mentally represented an object if they can correctly detect *matches and mismatches* to the object at a later time” (Baars, 2011, p. 41)^16^. It is also unclear what criteria have to be satisfied for being a representational state, uncertain “just how completely cognitive states must be representational. Must every component of every cognitive state be a representation?” (Adams and Aizawa, 2010, p. 55). Tinging the curious position of representation as used in science, we can add that it is admitted that we do not know any physiological marker which can indicate that a mental representation has been formed (Frith, 1999, p. 106).

Subsequently I will list some examples of different usages of the term *representation*. This list clearly demonstrates that beyond a polymorphic character^17^ of the term, its usage raises numerous additional questions to be answered. Even the demarcation in the usage of public and mental representation is not as simple as would be expected.

(1) We can read about *conscious representation*, although “[w]e still do not understand exactly how millions of neuronal discharges, distributed across time and space, encode a conscious representation”. As far as I know, the attempt to find “new mathematical instruments in order to understand these complicated patterns” is still a vivid endeavor (Dehaene, 2014, p. 164f).
(2) We know there are *unconscious representations* as well: the bottleneck of consciousness suggests we have unconscious mental representations competing to become conscious ones (Dehaene, 2014, p. 46). But, because unconscious mental representations are not accessible subjectively, it does not help us find out what we should think about these unconscious representations. Therefore, when we detect them we can have only indirect traces, if any at all.
(3) “When I look at a tree in the garden, I don’t have the tree in my mind. What I have in mind is a model (or representation) of the tree constructed by my brain. This model is built up through a series of guesses and predictions” (Frith, 2007, p. 170). That is, the *model* of a tree as a mental representation is the result of the model-making brain’s learning process.
(4) As conscious percepts, ideas also have a *neural activation pattern*. As Baars and Gage (2013) proposed: “Ideas appear to be represented in the cortex in terms of complex webs of learned connectivities, rather than localized filing systems with neatly arranged conceptual categories” (2013: 360; emphasis added).
(5) Neural activation patterns as representations of conscious percepts seems to be even more puzzling if we take into account the working of certain *specialized neurons*^18^.

The above listed usages apparently suggest at least two distinct senses: (i) in the sense of *neural pattern*, i.e., as a mental event described in terms of neuronal activity; and (ii) in the sense of *the content of consciousness*, as something that usually can be reported and “refers to a mental entity that stands for something in the external world” (Frith, 1999, p. 107), or as Baars (2011) suggested, a theoretical construct that stands for a mental phenomenon.

In the first case, representation is publicly available, i.e., we have access to neural states via brain imaging technologies. However, the neural representation of a mental state raises the question ‘‘What does a mental state consists of?’’ If it is the result of a certain neuronal pattern, then we can consider it being the cause of the state. Hence, the representation of the neural pattern is basically the representation of the cause of the given state. If we think that a mental state consists in/of certain neuronal patterns, then the reconstructed image of it is a public representation. But we have to account for other expressions of the given mental state, i.e., its bodily and/or linguistic expression^19^.

In the second case we should ask “How we can have access to the content of consciousness at a neuronal level?” As Dehaene suggested we cannot yet crack the code of how the brain creates a mental state (Dehaene, 2014, p. 179), and we do not know any physiological markers which can indicate that a mental representation has been formed (Frith, 1999, p. 106). Therefore, we hardly have any access to the content of consciousness as it emerges in the neural network, it seems to be merely presupposed. That is, mental representation stands for something, it signifies a content to which we have no access – except if we account for the bodily or linguistic expression of it. However, we can ask along with James whether mental representation as conscious content isn’t a duplicate of the same phenomenon (James, 1987, p. 1151).

We have no evidence that cognitive processes involve mental representation, it is merely presupposed; and there is no provable criterion of the becoming of a mental representation in neural activity. We have direct access only to the scenery, behavior, report, and recorded neural states. In accordance with this information, *mental representation* was introduced as a shortening of what could be the content of the conscious experience and/or cognitive state of which we can find the neural correlate. But the extensive use of the term suggests there is something beyond the neural correlates and the bodily and linguistic expression that is to be found a “*gaseous thing*” (Wittgenstein, 1979, p. 31f) in terms of physical phenomena. The use of the term mental representation promises something that originally was not intended.

### Representation and Embodied Mind

Recent work in embodied cognition sheds some new light on the role of representation. Varela et al. (1993, p. 9) in their programmatic book suggest “that cognition is not the representation of a pregiven world by a pregiven mind but is rather the enactment of a world and a mind on the basis of a history of a variety of actions that a being in the world performs.” Some approaches of embodied cognition do not call into question, while others demand a dramatic decrease in the role mental representation plays in psychology and cognitive science. Taking into account their predecessors’ suspicion with regard to representation, we can see they were aware of the limits of science: science is to go into details as opposed to the whole (Bergson, 1946), it is to analyze, therefore it separates and dissects the investigated phenomenon, unintentionally distorting the studied mechanism or organism (Merleau-Ponty, 1967, p. 216).

The idea of looking for influential factors beyond the realm of mental representations as thought-shapers is parallel with the idea of understanding cognition beyond brain-bound processes. Representational means as a facilitator and definer of the expressible, and therefore shaper of the epistemic accessible, are decisive. At the beginning of the so-called secondary orality, Bergson aptly described the peculiarities of concept formation as well as the ambiguities raised by the term *idea* and later *representation*, clearly demonstrating how difficult it is to comprehend mentality. These insights suggest that disorienting concepts make us ask questions that are unanswerable despite the opportunities provided by sophisticated technical devices.

## Conclusion

Representational skills paved the way to creating new representational means and systems. This intertwined relation of cognitive and external/technical facilities introduced a new attitude toward the world: science became an increasingly specialized enterprise with testable methodology to capture the different segments of reality. It is considered as an ever progressive enterprise with reliable, mostly not counterintuitive results. But in the meanwhile, it goes largely ignored that this reliability is only partial – that evidence is valid only within a well-defined framework; the access to the investigated part of the world is decisively influenced by the theory by which we attempt to illuminate further details, i.e., we are basically biased by the applied theory and by the applied methods and means since these determine what is accessible and imaginable for us. As we can see, the understanding and apprehension of the world or a particular phenomenon is decisively defined by the means we use for description and/or depiction. If we can use only words as definitions, particulars are not considered; if particulars are duplicable, we face the dilemma of whether general features or particular cases or rather structural relations are favorable. Beyond these biasing factors, there is an even more encompassing deceptive mediator of thought: language. As it evolved and became increasingly refined in accordance with the needs of different areas of interest, it suggested a structure of reality akin to its own nature, viz., differentiating and at the same time generalizing, highly abstract though metaphoric. Though the universe of concepts refers to the world of things, it is expected to provide access to mental phenomena as well.

## Author Contributions

The author confirms being the sole contributor of this work and has approved it for publication.

## Conflict of Interest

The author declares that the research was conducted in the absence of any commercial or financial relationships that could be construed as a potential conflict of interest.

## Publisher’s Note

All claims expressed in this article are solely those of the authors and do not necessarily represent those of their affiliated organizations, or those of the publisher, the editors and the reviewers. Any product that may be evaluated in this article, or claim that may be made by its manufacturer, is not guaranteed or endorsed by the publisher.

## Footnotes

1 Affordances are environmental qualities understood in relational terms regarding the living organism. In accordance with the structural features of the environment and/or the organism’s sensorimotor capabilities, certain features of the environment are recognizable and exploitable. The term reaches back to the Gibsonian ecological approach.

2 The enactive approach is a significant view in the field of embodied cognition. It suggests that “living beings are autonomous agents that actively generate and maintain themselves, and thereby also enact or bring forth their own cognitive domain” (Thompson, 2007, p. 13). For more details see Varela et al. (1993) and Thompson (2007).

3 In the so-called “verbomotor” cultures we can notice a balanced symmetry of the human body and kinesthesia and the recitation of prayer. “Such balanced structures are designed to be retained by being recited while one rocks back and forth, at least in imagination, as the Koran is retained in still highly oral Arab societies” (Ong, 1981, p. 30).

4 We should note here, that while pictures played an important role in the development of science, according to the Calvinist assumption “[t]he illiterate should not be given images but should be taught to read”. That is, in a wider context we can recognize a “movement from image culture to word culture” (Eisenstein, 1979, p. 67).

5 “The only knowledges in which the Greeks made great advances were geometry and astronomy, for the first of which words amply suffice, and for the second of which every clear night provides the necessary invariant image to all the world” (Ivins, 1953, p. 16). As generalities we can think of “Plato’s Ideas and Aristotle’s forms, essences” and “the well-known notions of substance and attributable qualities” (Ivins, 1953, p. 63). The question of generalization will be discussed in more detail in section “Language Spatialized.”

6 “It is precisely this close fit between techniques and practices that supplies the rationale for the at-first-glance-roundabout strategy of studying notions as abstract as truth and objectivity through concrete ways of making images for scientific atlases” (Daston and Galison, 2007, p. 41).

7 “In the course of Western history, *logos* changes from the event of the manifestation of beings to an instrument by which man gains control over the forces of nature” (Zimmermann, 1981, p. 220).

8 A fragment around 400 B.C. suggested some methods for how to learn and memorize: pay attention, repeat, and relate to known items. In the last case, memory of things and memory of names has been differentiated but in both cases images played an important role (Yates, 1984, p. 30).

9 Plato expressed the importance of sight quite explicitly: In the *Republic* he wrote “an eye of the soul is more precious far than ten thousand bodily eyes” because truth is visible only by the eye of the soul (Plato, 1997, *Republic* 527d-e); and in *Timaeus* we can read that “sight … is the source of the greatest benefit to us” since without it we could not know even the words by which we describe the universe (Plato, 1997, *Timaeus* 41a).

10 According to Heidegger, the difference between calculative and meditative thinking describes a basic difference in attitude toward the things around us. The former is perpetually calculating, like computers, it plans and envisions expected results, takes into account all parameters, and “races from one prospect to the next.” We would say it is a more and more specific feature of science. But it is rather far from the primordial human way of thinking, which “contemplates the meaning which reigns in everything that is” (Heidegger, 1966, p. 46), a patient and receptive mental disposition (Gelassenheit).

11 It is beyond the scope of the present paper, but worth noting that even the meaning of space has altered dramatically in the time of literacy. If we study images before the invention of the printing press, we can notice that space in medieval times was filled differently: the arrangement of the scenery was in accordance with importance, independently of the outlook of the depicted situation. With the appearance of the *new art*, spatial organization started to be arranged in accordance with the depicted scenery, i.e., the earlier important features of protagonists (their size and location in accordance with their position and importance) resigned its place to the rules of perspective painting and visual delight. For more details see Engström and Selinger (2009) and Kondor (2013).

12 I have to note here that conceptual metaphor theory dedicates an important role to the body in concept-formation. It suggests body and bodily engagements provide the ground for meaning (Johnson, 1990). In the present paper, however, I will not recapitulate how cross-domain mapping is based on bodily experiences and can result in abstract terms, but rather, I will focus on Bergson’s theory. I decided to refer basically to Bergson because (i) his remarks on language and metaphysics fit very well with the idea that language has considerable thought-shaping potential; (ii) Bergson’s theory is very close to the idea of the embodiment; (iii) many of his suggestions are recognizable among the ideas of Wittgenstein and Merleau-Ponty; and (iv) his theory includes important elements helpful to an understanding of ocular-centrism and spatially oriented concept formation.

13 As a margin note makes clear, “the distinction between mind and matter should be made in terms not of space, but of time or duration, which admits of degrees” (Bergson, 1911, p. 295), i.e., he did not want to eliminate dualism, rather he suggested a framework within which mind and matter pervade each other gradually. His solution is not as clear and undoubtful as we would expect. But, according to his reconstruction, we can get from matter to spirit then back to matter if we start with perception and accept that “the humblest function of spirit is to bind together the successive moments of the duration of things” (Bergson, 1911, p. 296).

14 The clarification of how consciousness and spirit relate to each other is far beyond this paper’s scope. However, Bergson bestows not only humans, but also matter with consciousness to a certain extent. For details see Bergson (1911, p. 313f) and Bergson (1946, p. 35).

15 It would far exceed the scope of the present paper to examine in detail Wittgenstein’s remarks on language. For details see Kondor (2015, p. 142–144).

16 Cf., “We would not want to claim that these neurons (in different visual areas which participate in creating the visual field of an organism) are representations simply by virtue of their causal connections to environmental stimuli, but we do think they are *likely to turn out to be representations*. These considerations seem to us to provide defeasible reasons to accept what cognitive *psychologists typically presuppose*, namely that cognitive processes involve representations” (Adams and Aizawa, 2010, p. 33; emphasis added).

17 For details see Kondor (2015, p. 142f).

18 There are single neurons which can be activated by a definite picture, sound, etc., but only in the case of conscious perception. For details see Dehaene (2014, p. 172f).

19 See James (1884, p. 189f) and Wittgenstein (1958, p. 41f).
